# Combination Treatment with MEK and AKT Inhibitors Is More Effective than Each Drug Alone in Human Non-Small Cell Lung Cancer *In Vitro* and *In Vivo*

**DOI:** 10.1371/journal.pone.0014124

**Published:** 2010-11-29

**Authors:** Jieru Meng, Bingbing Dai, Bingliang Fang, B. Nebiyou Bekele, William G. Bornmann, Duoli Sun, Zhenghong Peng, Roy S. Herbst, Vassiliki Papadimitrakopoulou, John D. Minna, Michael Peyton, Jack A. Roth

**Affiliations:** 1 Section of Thoracic Molecular Oncology, Department of Thoracic and Cardiovascular Surgery, The University of Texas MD Anderson Cancer Center, Houston, Texas, United States of America; 2 Department of Biostatistics, The University of Texas MD Anderson Cancer Center, Houston, Texas, United States of America; 3 Department of Experimental Therapeutics, The University of Texas MD Anderson Cancer Center, Houston, Texas, United States of America; 4 Department of Thoracic/Head & Neck Medical Oncology, The University of Texas MD Anderson Cancer Center, Houston, Texas, United States of America; 5 Hamon Center for Therapeutic Oncology Research, and Simmons Cancer Center, The University of Texas Southwestern Medical Center at Dallas, Texas, United States of America; National Cancer Institute, United States of America

## Abstract

AZD6244 and MK2206 are targeted small-molecule drugs that inhibit MEK and AKT respectively. The efficacy of this combination in lung cancer is unknown. Our previous work showed the importance of activated AKT in mediating resistance of non-small cell lung cancer (NSCLC) to AZD6244. Thus we hypothesized that dual inhibition of both downstream MEK and AKT pathways would induce synergistic antitumor activity. In this study, we evaluated the efficacy of AZD6244 and MK2206 individually on a large panel of lung cancer cell lines. Then, we treated 28 human lung cancer cell lines with a combination of AZD6244 and MK2206 at clinically applicable drug molar ratios. The AZD6244-MK2206 combination therapy resulted in a synergistic effect on inhibition of lung cancer cell growth compared to the results of single drug treatment alone. MK2206 enhanced AZD6244-induced Bim overexpression and apoptosis in A549 and H157 cells. When we tested the combination of AZD6244 and MK2206 at ratios of 8∶1, 4∶1, 2∶1, and 1∶8, we found that the synergistic effect of the combination therapy was ratio-dependent. At ratios of 8∶1, 4∶1, and 2∶1, the drug combination consistently demonstrated synergy, whereas decreasing the ratio to 1∶8 resulted in a loss of synergy and produced an additive or antagonistic effect in most cell lines. Furthermore, the AZD6244-MK2206 combination therapy showed synergy in the suppression of A549 and H157 xenograft tumor growth and increased mean animal survival time. The AZD6244-MK2206 combination therapy resulted in effective inhibition of both p-ERK and p-AKT expression in tumor tissue. In addition, a significant increase of apoptosis was detected in tumor tissue from mice treated with AZD6244-MK2206 compared with that from the single agent treated mice. Our study suggests that the combination of AZD6244 and MK2206 has a significant synergistic effect on tumor growth *in vitro* and *in vivo* and leads to increased survival rates in mice bearing highly aggressive human lung tumors.

## Introduction

The phosphatidylinositol 3-kinase (PI3K)/Akt and RAS/RAF/mitogen-activated protein kinase (MEK)/extracellular signal-regulated kinase (ERK) pathways, mediate proliferation and survival in human lung cancer cells and share several downstream molecules, such as FOXO3a [1], caspase-9 [2], and Bad [3].

Currently, a wide range of small-molecule tyrosine kinase inhibitors that target signaling pathways have been developed, and two of these agents are currently being evaluated in clinical trials. AZD6244 is an allosteric inhibitor of the MEK1/2 kinases that does not compete with adenosine triphosphate (ATP) binding activity [4]. This compound binds to MEK1/2 and induces several conformational changes in the unphosphorylated MEK1/2 enzymes, inhibiting their catalytic activity, which results in an inhibition of ERK activation and a blockade of the signal transduction pathways. MK2206 is a highly selective non-ATP competitive allosteric inhibitor of AKT with IC_50_ in the nM range and has broad preclinical antitumor activity. It is also in early phase clinical trials and is being evaluated in the treatment of patients with lung cancer. However, the potential efficacy of a combination of AZD6244 and MK2206 in the treatment of lung cancer is unknown. In this study, we investigated the effect of the combination of AZD6244 and MK2206 in killing human lung cancer cell lines and found that this combination was highly synergistic *in vitro* and very effective in the treatment of lung cancer xenografts. We also explored the mechanism of synergism for these two compounds. Our preclinical findings support clinical investigations of AZD6244 and MK2206 combination therapy in lung cancer patients.

## Materials and Methods

### Materials

AZD6244 and MK2206, synthesized in Dr. William G. Bornmann's laboratory at The University of Texas MD Anderson Cancer Center, were dissolved to concentrations of 25 mM and 20 mM, respectively, in dimethyl sulfoxide and stored at −80°C. Antibodies against total and phosphorylated ERK and AKT were purchased from Cell Signaling Technology (Danvers, MA). Antibodies against Bim were obtained from Calbiochem (San Diego, CA). Protease inhibitor cocktail, β-actin antibody, and sulforhodamine B were obtained from Sigma Chemical Corporation (St. Louis, MO). Protein assay materials were purchased from Bio-Rad Laboratories (Hercules, CA). DeadEnd™ Flurometic TUNEL System was purchased from Promega (Madison, WI).

### Cell culture

All the human lung cancer cell lines were provided by either Dr. John V. Heymach at MD Anderson Cancer Center or Drs. Adi Gazdar and John D. Minna at The University of Texas Southwestern Medical Center at Dallas. The cell lines were maintained in RPMI 1640 or high-glucose Dulbecco's modified Eagle's medium (DMEM), supplemented with 10% fetal bovine serum, 100 µg/mL ampicillin, and 0.1 mg/mL streptomycin; the cells were cultured at 37°C in a humidified atmosphere containing 5% CO_2_ and 95% air.

### Cell viability assay

The inhibitory effects of AZD6244, MK2206, and the combination of AZD6244 and MK2206 on cell growth were determined by using the sulforhodamine B assay, as described previously [5]. Each experiment was performed in quadruplicate and repeated at least three times. The relative cell viability (%) was calculated using the equation OD_T_/OD_C_×100% (where OD_T_ represents the absorbance of the treatment group and OD_C_ represents the absorbance of the control group). The median inhibitory concentration (IC_50_) values were determined using CurveExpert 1.3 software and plotted in dose-response curves.

### Western blot analysis

Whole-cell lysates were prepared by washing the cells with phosphate buffered saline (PBS) and subjecting them to lysis with Laemmli sample buffer supplemented with the protease inhibitor cocktail. After the lysates were sonicated for 15 s, the protein concentrations were quantified using the Bio-Rad protein assay kit. Equivalent amounts of each protein were loaded, separated by 10% or 12% sodium dodecyl sulfate-polyacrylamide gel electrophoresis, and then transferred to Polyvinylidene Fluoride (PVDF) membranes at 80 V for 2 h. The membranes were blocked for 1 h with 5% nonfat dried milk in PBS buffer containing 0.1% Tween-20 (PBST) and probed with diluted primary antibody at 4°C overnight. The membranes were then washed three times in the PBST buffer and probed with infrared dye-labeled secondary antibodies; the immunoreactive bands were visualized with the Odyssey Imager (Li-COR Biosciences, Lincoln, NE).

### Cell cycle and apoptosis assays

The cells were harvested by trypsinization, washed twice in cold PBS, fixed with ice cold 70% methanol, and incubated at 4°C overnight. Cells were then washed with PBS and incubated with 25 µg/mL propidium iodide containing 30 µg/mL ribonuclease for 30 min at room temperature. Cells were analyzed on an EPICS Profile II flow cytometer (Coulter Corp., Hialeah, FL) using the Multicycle AV software (Phoenix Flow Systems, San Diego, CA). Experiments were repeated at least three times.

### Animal studies

All Animal experiments were carried out after approval by the MD Anderson institutional review board (11-03-09932) and were performed in accordance with the Guidelines for the Care and Use of Laboratory Animals published by the National Institutes of Health.

Female BALB/c nude mice were purchased from Charles River Laboratories (Wilmington, MA). The mice were housed in laminar flow cabinets under specific pathogen-free conditions and were used when they were 6- to 8-weeks old. A total of 3×10^6^ H157 or A549 cells were inoculated subcutaneously into the right dorsal flanks of the nude mice. When the tumors reached an average volume of about 0.1 cm^3^, the mice were randomly divided into control and treatment groups (*n* = 5 animals per group). For the H157-bearing mice, the treatment groups were administered 20 mg/kg AZD6244, 10 mg/kg MK2206, or AZD6244-MK2206 combination at 20mg/kg–10mg/kg, all of which had been solubilized in a medium containing 0.5% hydroxypropyl methylcellulose and 0.1% polysorbate buffer. In the A549-bearing mice, the treatment groups were administered 24 mg/kg AZD6244, 6 mg/kg MK2206, or AZD6244-MK2206 combination at 24mg/kg–6mg/kg, all of which had been solubilized in a medium containing 0.5% hydroxypropyl methylcellulose and 0.1% polysorbate buffer. All drugs were dissolved in 100µl vehicle buffer for each mouse. All drugs administered twice daily by oral gavage. The control group received the vehicle buffer alone. The treatment duration was 20 d. Tumor size, measured by calipers, was recorded every 5 d. The tumor volume was calculated, taking length to be the longest diameter across the tumor and width to be the corresponding perpendicular diameter, using the following formula: length×width^2^×0.52. The tumor growth inhibition rate was calculated as 100%×(tumor size_treated_/tumor size_control_) on each measurement day. Tumor-bearing mice continued treatment as indicated above after 20 d. Mice were allowed to live up to their natural death or were sacrificed when their tumor volume was larger than 2000 mm^3^. Kaplan-Meier survival curves were plotted and statistically analyzed. Animal body weight was measured and recorded every 5 d during the treatment.

For the pharmacodynamic study, tumors were established as described above and were allowed to grow to a size of 0.5–0.8cm^3^ before treatment started. Mice bearing s.c. A549 tumors were then daily administered vehicle, AZD6244, MK2206 or AZD6244-MK2206 at the same concentrations mentioned above (*n* = 5 animals per group) for 3 days. Four hours after the last dose, animals were euthanized and tumors were resected, fixed with 4% paraformaldehyde and paraffin-embedded for immunohistochemistry staining and TUNEL assay.

### Immunohistochemistry

The sections were deparaffinized in xylene and serial ethanol dilutions. The antigens were retrieved and endogenous peroxidase activity was blocked with 0.3% hydrogen peroxide for 10 min. The sections were then treated with 10% normal goat or horse serum for 30 min. After overnight incubation with primary antibodies, including p-AKT (dilution 1∶100) and p-ERK (dilution 1∶100), the sections were probed with biotinylated secondary antibodies and then incubated with streptavidin-biotin-complex (Lab Vision, Fremont, CA). The sections were then stained with a solution of 3,3-diaminobenzidine tetrahydrochloride (DAB; Lab Vision, Fremont, CA), counterstained with Mayer's haematoxylin (Fisher Scientific, Pittsburgh, PA), dehydrated and mounted.

### TUNEL assay

The sections were deparaffinized and the TUNEL assay was performed following the manufacturer's instructions of a commercially available kit (DeadEnd™ Fluorometric TUNEL System) from Promega. Apoptotic cells exhibit a strong nuclear green fluorescence that could be detected using a standard fluorescein filter. All cells stained with DAPI exhibit a strong blue nuclear fluorescence. The slides were observed under fluorescence microscopy with relative apoptotic cells determined by counting TUNEL-positive cells in five random fields (at ×100 magnification) for each sample.

### Statistical analysis

The *in vitro* cytotoxicity experiments were performed in triplicate for each time point and concentration. The significance of the *in vitro* data was determined using the Student *t* test (2-tailed). P-values less than 0.05 were considered statistically significant.

The gene mutations of the cell lines were obtained from online database (http://www.sanger.ac.uk/genetics/CGP/CellLines/). The correlations of the *in vitro* response to AZD6244 or MK2206 (IC_50_) and gene mutations were assessed by using Wilcoxon test and Logistic regression.

For the *in vivo* studies, tumor volumes were calculated as mean ± standard deviation. Wilcoxon rank sum test and Kruskal-Wallis test were used to compare treatment differences. Spearman correlation coefficient was used to estimate the correlation between two continuous variables. Treatment differences with respect to survival were assessed via the log-rank test. All tests were two-sided. P-values less than 0.05 were considered statistically significant. Statistical software SAS 9.1.3 and S-PLUS 8.0 were used for all the analyses.

## Results

### Effects of AZD6244 or MK2206 Single-drug Treatment on Lung Cancer Cell Lines

**Figure 1:**
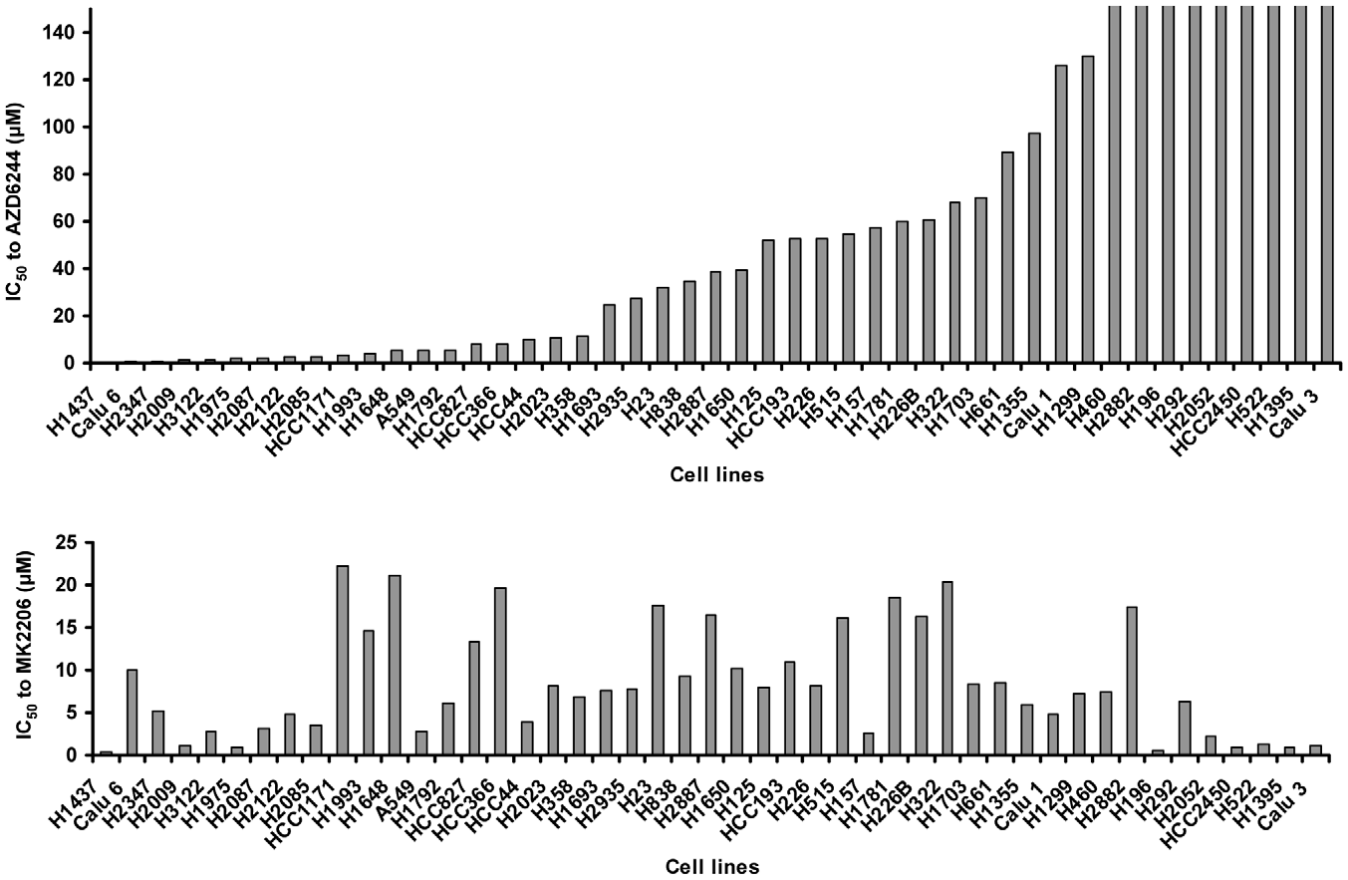
Response to AZD6244 or MK2206 alone in various lung cancer cell lines. The indicated cell lines were treated with different concentrations of either AZD6244 or MK2206 for 96 h. Cell viability was determined using sulforhodamine B, and IC_50_ was calculated according to the dose-response curve.

Before evaluating the effects of the combination therapy, we tested the antiproliferative effect of AZD6244 and MK2206 as single-agent therapies on a panel of 47 human lung cancer cell lines (Figure 1). The response to AZD6244 varied greatly, from highly sensitive (IC_50_<0.2 µM) to highly resistant (IC_50_>150 µM), among the cell lines. The dose response range of MK2206 was more consistent, with the IC_50_ ranging from 0.4 µM to 25 µM. We found some cell lines were sensitive to AZD6244 but resistant to MK2206 (such as Calu-6, HCC1171, and H1993), while other cell lines were resistant to AZD6244 but sensitive to MK2206 (such as H522, H1395, and Calu-3). No correlation between the sensitivity to these two compounds was observed. Comparing the dose-response results and the online gene mutation database, no correlation was found between EGFR, KRAS, BRAF or PI3K gene mutations and the IC_50_ of AZD6244 or MK2206. We also did not observe correlations between overall EGFR/KRAS/BRAF gene mutations and the IC_50_ of AZD6244 or overall EGFR/PI3K gene mutations and the IC_50_ of MK2206 (Table 1 and 2).

**Table 1:** Correlations between the IC_50_ to AZD6244 and gene mutations in lung cancer cell lines.

Gene mutation	Wilcoxon Test		Logistic Regression	
	P-value	Significance	P-value	Significance
EGFR	0.6493	NS	0.4631	NS
KRAS	0.2740	NS	0.2871	NS
BRAF	0.8513	NS	0.3527	NS
PI3K	0.2973	NS	0.2874	NS
EGFR/KRAS/BRAF	0.2667	NS	0.3595	NS

S = Significant.

NS = No Significant.

**Table 2:** Correlations between the IC_50_ to MK2206 and gene mutations in lung cancer cell lines.

Gene mutation	Wilcoxon Test		Logistic Regression	
	P-value	Significance	P-value	Significance
EGFR	0.2319	NS	0.4209	NS
KRAS	0.3206	NS	0.2710	NS
BRAF	0.1191	NS	0.2165	NS
PI3K	1.0000	NS	0.8898	NS
EGFR/PI3K	0.3117	NS	0.5499	NS

S = Significant.

NS = No Significant.

### Effects of the AZD6244-MK2206 combination vary among lung cancer cell lines

**Figure 2:**
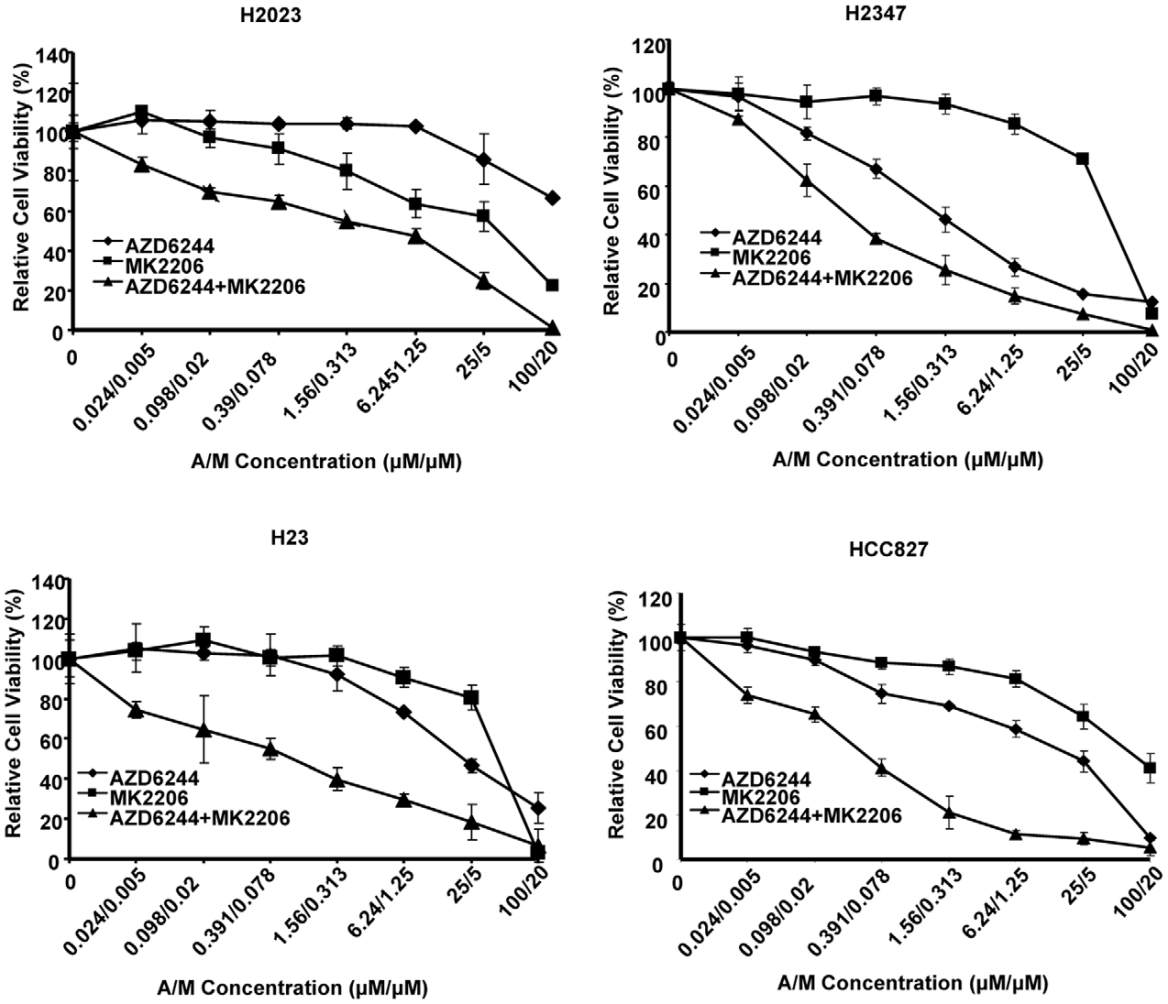
Dose-effect curves for the AZD6244-MK2206 combination for lung cancer cell lines. Lung cancer cell lines were treated with various concentrations of AZD6244 (0.024–100 µM), MK2206 (0.005–20 µM) and AZD6244/MK2206 (0.024/0.005–100/20 µM) for 96 h. Dose-effect curves of the combination and of each agent alone are presented for comparison. Representative cell lines that demonstrated the strong synergistic effects of the combination therapy are shown.

From the 47 cell lines, we chose 28 to test the antitumor effects of AZD6244 and MK2206 combination therapy. The cell lines were selected to represent a spectrum of sensitivity to one or both drugs. The IC_50_ was >5uM for both AZD6244 and MK2206 for 21 of the 28 cell lines. For the other cell lines the IC_50_ was <5uM for AZD6244 or MK2206 or both. To determine if the antitumor effects obtained with different AZD6244 and MK2206 combinations were synergistic, we calculated the combination index (CI) according to the Chou-Talalay method using Calculsyn software (Biosoft, Cambridge, UK). (CI>1.1, antagonism; CI = 0.9–1.1, additive effect; CI = 0.2–0.9, synergism; CI<0.2 strong synergism). Since the Chou-Talalay model calls for cytotoxic agents to be used at a fixed dose ratio, we chose to use AZD6244 and MK2206 at a 5∶1 molar ratio. After treatment with various concentrations of AZD6244 (0.024–100 µM), MK2206 (0.005–20 µM) and AZD6244/MK2206 (0.024/0.005–100/20 µM), which are in the range of concentrations achieved in the serum of patients receiving oral AZD6244 and MK2206, the combination index (CI) was measured on each cell line. In 67% of the cell lines, including H2023, H2347, HCC827, and H23 shown in Figure 2, the combination treatment produced a strong synergistic effect. The combined treatment produced an additive or antagonistic effect only in 11% of the cell lines (Table 3).

**Table 3:** Combination index (CI) value of the combination therapy of AZD6244 and MK2206 at the ratio of 5∶1.

Cell Lines	50% CI	75% CI
H23	0.03	0.19
HCC193	0.05	0.03
HCC827	0.05	0.06
H2023	0.06	0.13
H1693	0.06	0.17
H1299	0.06	0.02
H1703	0.06	0.01
HCC44	0.09	0.15
H1993	0.09	0.17
H125	0.12	0.06
A549	0.12	0.32
Calu1	0.13	0.10
H661	0.13	0.20
H515	0.16	0.22
H2347	0.17	0.14
H3122	0.2	0.28
H157	0.24	0.19
H226	0.24	1.41
H1792	0.24	0.48
H460	0.31	0.42
H2009	0.45	0.44
H1650	0.52	0.57
H2882	0.67	0.81
H838	0.80	0.20
H1975	0.85	0.39
H196	0.97	1.44
H522	1.01	0.88
Calu6	2.48	1.30

### Synergistic effect of AZD6244-MK2006 combination therapy is ratio-dependent

**Figure 3:**
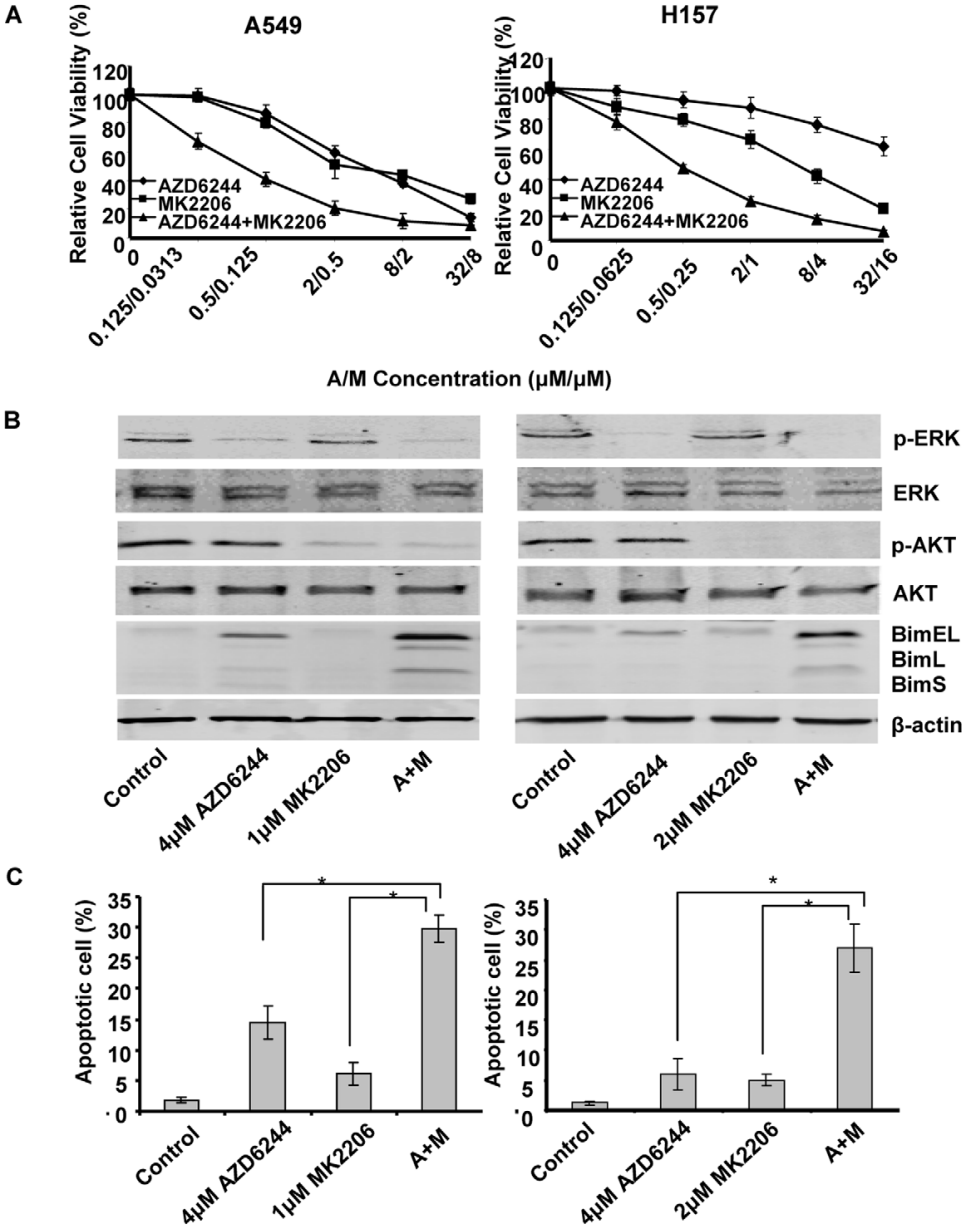
Treatment with AZD6244 and MK2206 synergistically upregulated Bim expression and induced cell apoptosis. (A) A549 and H157 were treated with AZD6244, MK2206, or a combination of these two compounds at the indicated concentrations for 48 h. (B) Protein specimens were harvested and AKT, p-AKT, ERK, p-ERK and Bim expression were detected with Western blot analysis. (C) Cells were trypsinized and fixed, and the cell cycle was measured with flow cytometry. Numbers represent percentages of apoptotic sub-G_1_–phase cells. Data represent one of three independent experiments with similar results. *Columns*, mean; *bar*, SD.*, *P*<0.05, compared with the single agent treatments.

The fixed drug ratios were expanded for 7 cell lines (H1792, H157, A549, H515, H1693, H1703 and H3122) that were sensitive to the combination of AZD6244 and MK2206 to 8∶1, 4∶1, 2∶1, 1∶2, 1∶4, and 1∶8. We found that consistent synergism resulted when the 8∶1, 4∶1, and 2∶1 ratios were used (Figure 2, Table 4), whereas decreasing the AZD6244∶MK2206 ratio to 1∶8 resulted in a loss of synergy and produced an additive or antagonistic effect in most cell lines (Table 4). We also found that each cell line had its own optimal drug ratio. For example, H1703 showed the highest level of synergism at an AZD6244∶MK2206 ratio of 8∶1; H1693 and A549 was highest at 4∶1(Figure 3A, left); and H157 was highest at 2∶1 (Figure 3A, right).

**Table 4:** Combination index (CI) value of the combination therapy of AZD6244 and MK2206 at the ratios of 8∶1, 4∶1, 2∶1, and 1∶8.

Cell Lines	8∶1		4∶1		2∶1		1∶8	
	50%CI	75%CI	50%CI	75%CI	50%CI	75%CI	50%CI	75%CI
H1792	0.1	0.26	0.22	0.1	0.39	0.83	1.01	1.03
H157	0.34	0.4	0.28	0.27	0.16	0.08	0.83	0.76
A549	0.26	0.39	0.1	0.12	0.21	0.24	0.99	0.79
H515	0.13	0.22	0.29	0.48	0.33	0.2	0.69	0.76
H1693	0.09	0.09	0.04	0.07	0.15	0.24	1.51	0.95
H1703	0.01	0.04	0.07	0.05	0.12	0.16	0.92	0.97
H3122	0.24	0.36	0.53	0.82	0.4	0.48	0.63	0.59

### MK2206 enhances the AZD6244-induced apoptosis

To determine if MK2206 had an effect on AZD6244-induced apoptosis, we tested the expression of AKT, p-AKT, ERK, p-ERK, and Bim and quantified apoptotic cells following treatment with individual and combination agents. AZD6244 is known to upregulate expression of Bim, a BH3-only protein, leading to a mitochondrial pathway activation and apoptosis, mediated by FOXO3a [6]. In addition, AKT can also phosphorylate FOXO3a, and inhibition of AKT might enhance FOXO3a activation by dephosphorylation. We noticed that treatment with AZD6244 alone led to a relatively moderate overexpression of Bim at 48 h. Although we did not detect an obvious change in Bim expression following treatment with MK2206, when MK2206 was combined with AZD6244, Bim expression increased to a level higher than that induced by AZD6244 alone (Figure 3B). We also found that the 2-drug combination resulted in more apoptotic cells than either single-drug treatment alone. Once combined with MK2206, AZD6244-induced apoptosis increased from 14.4% to 29.8% in the A549 cell line and from 6.0% to 27.0% in the H157 cell line (*P*<0.05, Figure 3C). These results indicate that MK2206 effectively enhanced AZD6244-induced activation of mitochondrial apoptosis.

### Synergistic effect *in vivo*

**Figure 4:**
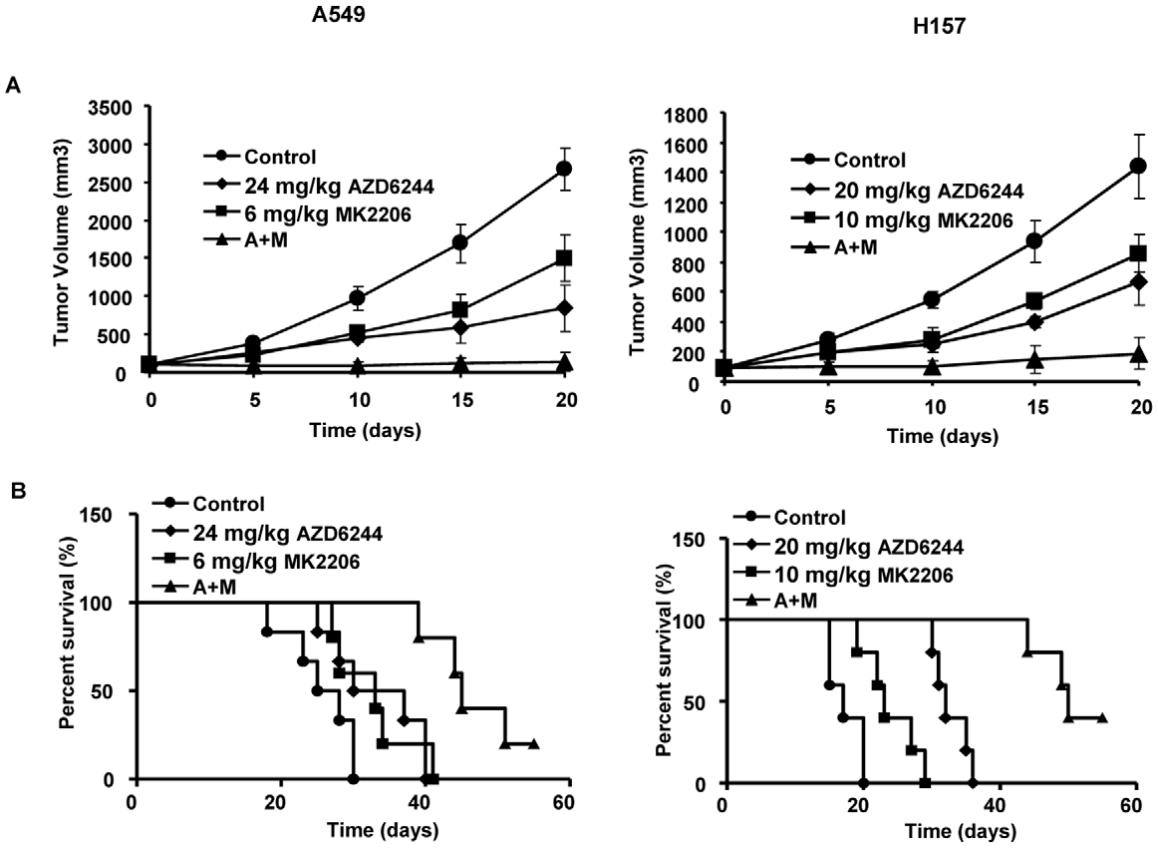
Effect of AZD6244-MK2206 combination on tumor growth and survival in mice. Flank tumors were established in nude mice and treated with vehicle, AZD6244, MK2206, or combination AZD6244-MK2206 therapy orally twice a day at indicated doses. (A) Tumor volumes. Overall differences between treatments were statistically significant (*P*<0.001; Kruskal-Wallis Test). We observed statistically significant time-by-treatment effects when comparing Control vs A (*P*<0.05); Control vs M (*P*<0.05); Control vs A+M (*P*<0.05); A vs M (*P* = 0.05); A vs A+M (*P* = 0.05) and M vs A+M (*P*<0.05) for A549; results were similar for H157 except that A vs. M was not statistically significant. (B) Survival times. Overall differences between treatments were statistically significant (*P*<0.001; Log-RankTest).

Response to the AZD6244-MK2206 combination treatment *in vivo* was evaluated in subcutaneous tumors generated by injection of A549 or H157 cells into the flanks of BALB/c nude mice. Mice with established flank tumors of equal volumes were treated with vehicle, a single AZD6244 administration, a single MK2206 administration, or the AZD6244-MK2206 combination. In both the A549 and H157 subcutaneous xenograft mouse models, mice receiving the combination of AZD6244 and MK2206 showed a significantly reduced mean tumor volume (135±120 and 188±107 mm^3^) compared with mice receiving AZD6244 alone (848±302 and 669±154 mm^3^), MK2206 alone (1497±380 and 858±125 mm^3^), or control treatment (2666±275 and 1437±217 mm^3^) by day 20 (*P*<0.01 for all three comparisons; Figure 4A). This result indicates that suppression of AKT with MK2206 increased the A549 and H157 cells' sensitivity to AZD6244 *in vivo*. In addition, we found that animal survival times were longer in the groups that received the 2-drug combination than in the groups that received single-agent compounds or control treatment (Figure 4B). The median survival time of animals treated with the AZD6244-MK2206 combination increased significantly (*P*<0.01, Figure 4B). In the A549 xenograft model, mice treated with AZD6244-MK2206 had a median survival time of 50 d, while those treated with AZD6244 alone, MK2206 alone, and control vehicle had median survival times of 32 d, 23 d, and 17 d, respectively (*P*<0.01 for all three comparisons). We had similar results in the H157 xenograft model: the median survival times for the combination therapy markedly increased to 45 d while for AZD6244-alone, MK2206-alone, and control treatments were 33.5 d, 33 d, and 26.5 d, respectively (*p*<0.01 for all three comparisons). In addition, 20% of the H157 tumor-bearing mice and 40% of A549 tumor-bearing mice that received the combination treatment survived past 55 d and did not have tumors at the final observation. Furthermore, no significant differences in mouse body weight were found between the four groups following the 20 d treatment, and there were no obvious toxicities from the drug combination (data not shown). Together, these results suggest that the combination of AZD6244 and MK2206 has a synergistic therapeutic effect on human lung cancer cell growth in a panel of NSCLC cell lines *in vitro*, and in A549 and H157 cell lines *in vivo*.

### Target modulation in A549 xenograft mouse model

**Figure 5:**
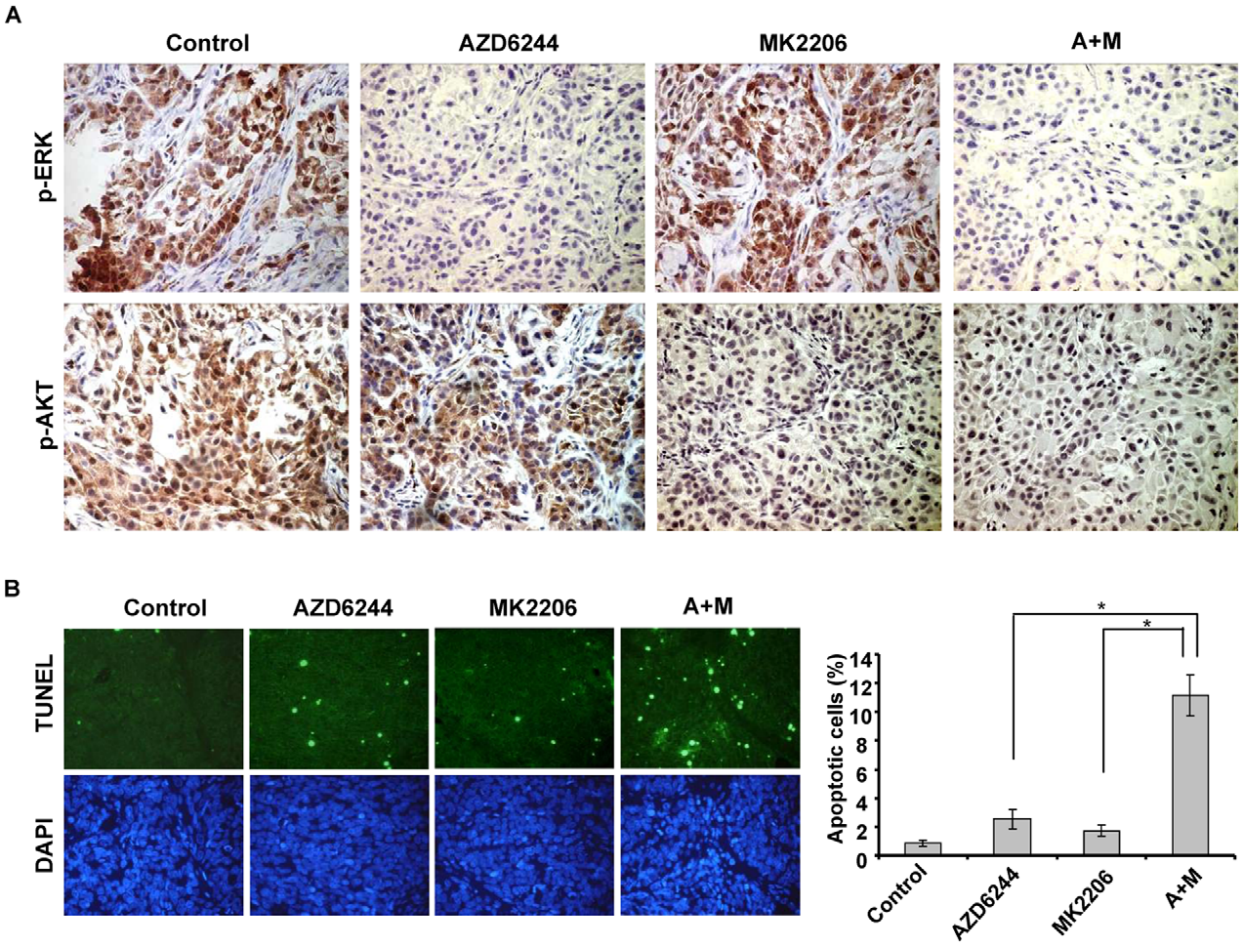
Pharmacodynamic effects of AZD6244-MK2206 on A549 xenograft mouse models. The xerograft mouse models were treated with vehicle control, AZD6244 (24 mg/kg), MK2206 (6 mg/kg) or AZD6244-MK2206 (24 mg/kg–6 mg/kg) combination for 3 days. Four hours after the last dose, the tumors were resected, fixed and paraffin-embedded. (A) The sections were analyzed by immunohistochemistry using p-ERK and p-AKT antibodies and the representative photographs in tumor sections are shown. (B) The sections were subjected to TUNEL and DAPI staining. The relative apoptotic cells were determined by counting TUNEL positive cells in five random fields (at 100× magnification) for each sample. *Columns*, mean; *bar*, SD. *, *P*<0.05, compared with the single agent treatments. The representative photographs in tumor sections are shown.

In the pharmacodynamic study, four hours after the final dose on day 3, the animals were euthanized and the tumors tissues were excised and analyzed for p-ERK and p-AKT by immunohistochemical staining. Inhibition of p-ERK expression was observed in tumors of mice treated with AZD6244 alone or the AZD6244-MK2206 combination. Inhibition of p-AKT was seen in tumors of mice treated with MK2206 alone or the AZD6244-MK2206 combination (Figure 5A). The results indicated that p-ERK and p-AKT were effectively inhibited with AZD6244 and MK2206 *in vivo*. The combination of AZD6244 and MK2206 could also inhibit the targets effectively. The TUNEL assay showed that MK2206 enhanced AZD6244-induced apoptosis significantly (*P*<0.05) from 2.6% to 11.2% in xenograft tumor tissue (Figure 5B).

## Discussion

In this study we report that the combination therapy of MEK inhibitor AZD6244 and AKT inhibitor MK2206 can induce dramatic synergistic inhibition of tumor growth *in vitro* and *in vivo*. Previously studies have observed that resistance to AZD6244 in lung cancer cells is mediated by AKT activation [5], [7]. The feedback loop of RAS/RAF/MEK/ERK and PI3K/AKT pathways prompted us to hypothesize that suppression of these two pathways may overcome the resistance to AZD6244 and act synergistically to inhibit the growth of lung cancer. In this study, we showed that single-agent therapy with either AZD6244 or MK2206 has a modest inhibitory effect on lung cancer cell lines. Because certain gene mutations may be implicated in response to these agents, we also checked the mutational status of BRAF, KRAS, EGFR and PI3K in these cell lines. The BRAF V600E mutation has been reported to be correlated with sensitivity to MEK inhibitors [8], [9] and has been used as a major criterion for recruiting patients into a clinical trial of MEK inhibitors in melanoma patients. KRAS, BRAF/KRAS, and LKB/KRAS mutation(s) are correlated with sensitivity to MEK inhibitors in ovarian cancer [10], colorectal cancer [11], and NSCLC [12], respectively. However, we did not detect an obvious correlation between mutational expression of EGFR, BRAF, PI3KI, or KRAS and sensitivity to AZD6244 or MK2206 in the lung cancer lines we tested. This may be due to the differing effects of the mutations in specific histologic types of cancer or in cell line specific pathways. For example, for breast and lung cancer, gene expression associated with differential sensitivity to AZD6244 included many genes that were not in common for both histologies [13]. Another possible explanation for the discrepancy of our results and previous studies could be due to various frequencies of gene mutations in different cancer types. For example, BRAF is mutated in many cancers, including malignant melanoma (27–70%), papillary thyroid cancer (36–53%), ovarian cancer (about 30%), and colorectal cancer (5–22%). However, BRAF mutations are detected only in 1–2% of lung cancer patients. It would be difficult to show statistically significant relationships with such low frequency events.

Our investigation showed that the dual-agent AZD6244-MK2206 combination therapy showed a synergistic effect on tumor growth relative to either agent alone. As a single-agent therapy, AZD6244 was ineffective against some lung cancer cell lines (such as Calu-1, H460, and H661). However, when combined with MK2206, these resistant cell lines were sensitive to combination treatment. According to Chou-Talalay methods [14], we used a fixed drug ratio to test for a synergistic effect. In clinical trials, the combination therapy will be given to patients in repeating 28-d cycles. The planned starting dose of MK2206 is 45 mg/kg every other day (the highest once every other day dose of MK2206 will be 45 mg/kg) or 90 mg/kg once weekly and increasing to as much as 250 mg/kg once weekly; the planned starting dose of AZD6244 is 75 mg/kg twice daily (http://clinicaltrials.gov/). We selected a drug ratio of 5∶1 AZD6244∶MK2206 which is in this range for our initial study *in vitro*. Our results showed that AZD6244∶MK2206 can induce synergistic effects among 89% cell lines. To obtain a more quantitative analysis, we expanded the drug ratios to identify the most effective range and the optimal synergistic ratio. We found that these two drugs showed consistently synergistic effects at 8∶1, 4∶1, and 2∶1 ratios of AZD6244 to MK2206, whereas decreasing this ratio to 1∶8 resulted in a loss of synergy and the presence of only additive effects or even antagonism in most cell lines. We then selected two KRAS mutated cell lines, A549 and H157, to further investigate the effect of AZD6244-MK2206 combination *in vivo*. The tumor growth was inhibited significantly with combination treatment compared to single agent treatment. The pharmacodynamic effects in A549 xerografts showed that the expression level of p-ERK and p-AKT were suppressed sufficiently with AZD6244 and MK2206 respectively. The combination of AZD6244 and MK2206 inhibited both p-ERK and p-AKT effectively. The TUNEL assay showed that combination treatment induced much more tumor cell apoptosis than individual drugs. These results strongly suggest that AZD6244 and MK2206 synergistically induce apoptosis by dual inhibition of ERK and AKT phosphorylation.

Previous studies have shown that whether anticancer drug combinations interact synergistically or antagonistically can depend on the ratio of the combined agents [15], [16]. Failure to control drug ratios because of uncoordinated mechanisms or pharmacokinetics could therefore lead to drug resistance if the tumor cells are exposed to antagonistic drug ratios. It is possible that the precise ratios described *in vitro* may not be obtained in the tumor of a patient due to the differential distribution and metabolism of these two compounds. However, we found that the synergistic effects occurred over a broad range of drug concentrations making it likely that a therapeutic effect will be achieved.

In our mechanistic and *in vivo* studies, we used A549 and H157 cell lines because both of them harbor KRAS mutations. Mutations in KRAS have been found in 20% to 30% of lung cancers and are believed to play a key role in this malignancy [17]. The presence of a mutated KRAS gene is reportedly associated with primary resistance to all targeted therapies [18]. The KRAS mutation also serves as a strong predictor of non-responsiveness to EGFR–targeted agents in lung and colon cancers [19]. Engelman and colleagues [20] recently reported that the combination of PI3K and MEK inhibitors led to marked synergistic tumor regression in KRAS-mutant mouse lung tumors. They later observed that an AKT inhibitor when combined with MEK inhibitors, failed to induce apoptosis in human lung cancer cells with mutant EGFR although these cells were sensitive to the combination of PI3K and MEK inhibitors [21]. However, in our study, we investigated that the combination therapy of MEK inhibitors and AKT inhibitor has a strongly synergistic effect on both EGFR mutant (such as HCC827) and EGFR wildtype cell lines. There are several possible reasons to explain this difference. Firstly, we used an allosteric inhibitor MK2206 in our study and they used an AKT1/2 inhibitor. The AKT1/2 inhibitor may not be as effective in inhibiting the three known isoenzymes of AKT thus resulting in a negative feedback loop which interferes with the efficacy of the combination therapy. Secondly, as the effect of the AZD6244-MK2206 combination is drug ratio-dependent, it is possible that the single drug ratio used in Engleman's study might not be the optimal ratio to induce synergy. Nevertheless, the efficacy of the combined suppression of ERK and AKT in KRAS-mutated cell lines confirm the strategy of dual downstream target inhibition converging on a common effector pathway, as previously reported by She et al [22], Legrier et al [23], Engelman et al [20] and Mordant et al [24]. This combination strategy represents a promising therapeutic strategy for tumors resistant to targeted therapies used as single agents.

In conclusion, our finding that the combination of AZD6244 and MK2206 results in a synergistic effect on inhibiting NSCLC cell growth and increasing survival times for mice bearing NSCLC xenografts may lead to an effective drug combination treatment strategy for lung cancer patients.
